# Application of Artificial Intelligence-Based Technologies in the Healthcare Industry: Opportunities and Challenges

**DOI:** 10.3390/ijerph18010271

**Published:** 2021-01-01

**Authors:** DonHee Lee, Seong No Yoon

**Affiliations:** 1 College of Business Administration, Inha University, Incheon 22212, Korea; dhlee04@inha.ac.kr; 2 Department of Business Edward Waters College, Jacksonville, FL 32209, USA

**Keywords:** AI-based technology, real-world cases, opportunities and challenges, policy and management support, healthcare industry

## Abstract

This study examines the current state of artificial intelligence (AI)-based technology applications and their impact on the healthcare industry. In addition to a thorough review of the literature, this study analyzed several real-world examples of AI applications in healthcare. The results indicate that major hospitals are, at present, using AI-enabled systems to augment medical staff in patient diagnosis and treatment activities for a wide range of diseases. In addition, AI systems are making an impact on improving the efficiency of nursing and managerial activities of hospitals. While AI is being embraced positively by healthcare providers, its applications provide both the utopian perspective (new opportunities) and the dystopian view (challenges to overcome). We discuss the details of those opportunities and challenges to provide a balanced view of the value of AI applications in healthcare. It is clear that rapid advances of AI and related technologies will help care providers create new value for their patients and improve the efficiency of their operational processes. Nevertheless, effective applications of AI will require effective planning and strategies to transform the entire care service and operations to reap the benefits of what technologies offer.

## 1. Introduction

Information and communication technology (ICT) is a core element of digitized organizations that can facilitate operational effectiveness and enhance competitive advantage [1,2,3,4]. In today’s Fourth Industrial Revolution (4IR) era, advanced digital technologies and devices are widely applied for innovation and value creation across industries [5]. The healthcare industry is no exception. Hospitals and care providers around the world, especially in developed economies, are aggressively deploying digital technologies, such as artificial intelligence (AI), machine learning, smart sensors and robots, big data analytics, and Internet of Things (IoT), for improved quality of care and operational efficiency [4]. A study by Aruba [6], a Hewlett-Packard Enterprise company, reported that more than 60% of hospitals worldwide have implemented IoT in their facilities. Therefore, it is valuable to investigate how advanced digital devices are affecting service encounters between customers and service providers in the healthcare industry [3,4].

Recently, there has been widespread applications of AI-supported technologies in healthcare institutions for improved care service quality and efficiency of medical resources [2,7]. Since AI encompasses machine learning, natural language processing, and smart robots, AI-based technologies provide numerous opportunities for innovation in the knowledge-intensive healthcare industry [8,9]. Dozens of startups, as well as existing image device companies that participated in the Radiological Society of North America (RSNA) conference held in Chicago in December 2018, made presentations on their AI initiatives that support accurate and reliable diagnosis and proper treatment of patients based on the data obtained from clinical examinations [10].

In addition, AI has attracted the attention of researchers, physicians, technology and program developers, and consumers in various fields in terms of its potential for transformative innovations in treating human diseases and public health [2,9,11,12]. According to Accenture [13], hospitals will invest $6.6 billion annually in AI-related technologies by 2021. Safavi and Kalis [9] (p. 1) estimate that “AI applications could create up to $150 billion in annual savings for U.S. healthcare by 2026.”

As AI-supported technologies learn and diagnose from a large volume of medical research and patients’ treatment records, they play a significant role in augmenting doctors’ decision-making process for diagnoses and treatment [14,15,16]. Shiraishi et al. [17] (p. 449) reported that “AI-based diagnostic algorithms are applied in the detection of breast cancer, serving as a ‘second opinion’ in assisting radiologists’ image interpretations.” It was also reported that AI technology can diagnose skin cancer more accurately than a professional dermatologist [18]. The diagnosis can be processed more quickly and efficiently because it is analyzed based on knowledge gained from a large body of knowledge and data [19]. Moreover, advanced virtual human avatars are being used to conduct conversations required to diagnose and treat patients with the mental disease [20].

Miyashita and Brady [21] provided an example of discharged patients who were fitted with a Wi-Fi-enabled armband that remotely monitors vital signs, such as respiratory rate, oxygen levels, pulse, blood pressure, and body temperature, from a group of hospitals serving 500,000 people in southeast England. In this case, hospital readmission rates and emergency room visits were reduced significantly through AI programs that analyze patient data in real-time. The need for expensive home visits was also reduced by 22%. In the long term, adherence to the treatment plan increased to 96% compared to the industry average of 50%. In another example, Grady Hospital, a public hospital in Atlanta, USA, reported $4 million in savings from a 31% reduction in readmission rates over two years due to the application of an AI-enabled tool to identify “at-risk” patients [21].

Considering such cases where AI serves a supporting/augmenting role in diagnosis and/or treatment and operation processes, some may assume that physicians will be rendered obsolete in the near future. However, it is necessary to first assess the role that AI can play to explore opportunities and challenges that are associated with AI applications in the healthcare industry. It is apparent, based on many real-world examples of AI applications, that AI has an enormous and wide-reaching potential with almost everything from simple operational process innovation to the most sophisticated treatments of emergency patients [9].

Some of the notable challenges involved in the widespread application of AI and digital devices include privacy concerns, cybersecurity, data integrity concerns, data ownership, the problem of data-sharing by various organizational silos, medical ethics issues, responsibility for medical errors, and risks of system failures [7,9,22]. Considering the nature of healthcare services, ethical issues are real challenges because AI technology may threaten patients’ preferences, safety, and privacy [2,7,19]. Currently, policies and ethical guidelines for healthcare services that incorporate AI and its applications lag behind the speed of advances in AI [19]. Also, AI-based technologies should encompass problem-solving flexibility and human-oriented values. However, AI-based technologies are still quite controversial in the healthcare industry because they are not yet universally available to all care providers. Therefore, there is a need to analyze existing cases of AI-based technologies and their applications to understand the future direction of their use in diagnoses, quality care services, and operational strategies.

Based on this context, this study analyzes several real-world examples in the healthcare industry to understand how AI affects care services and operational processes. This line of research will allow us to recommend a set of strategies to enhance the efficiency of patient treatment and prevention of diseases, as well as the operational efficiency of hospitals. For this purpose, we performed an extensive review of the literature and diverse real-world cases to uncover AI-based technologies and their applications in healthcare systems. This study is meaningful in that it presents new insights about the direction of technology-based service operations management. The results of our study are expected to provide valuable new information to hospital administrators, medical personnel, medical school curricula developers, education and training managers, human-machine roles and responsibilities specialists, privacy and cybersecurity analysts, and medical ethics professionals.

This paper is structured as follows. Section 2 presents a review of the relevant literature. Section 3 presents real-world cases featuring AI-based technologies. Section 4 discusses opportunities and challenges involved in applying AI-based technologies, and possible ways to prepare for the expanded application of advanced digital technologies in the healthcare industry. Section 5 concludes the study by summarizing the results, articulating the implications to the literature and practice, as well as its limitations and future research needs.

## 2. Review of Relevant Literature

**Figure 1:**
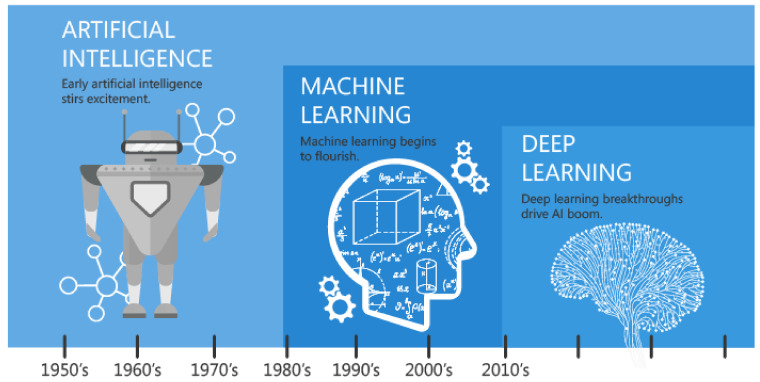
Evolution of Artificial Intelligence [23].

Artificial Intelligence (AI) refers to the simulation of human intelligence in machines like computers or robots that are programmed to mimic cognitive functions that humans associate with other human minds, such as learning and problem-solving. Artificial Intelligence, machine learning, and deep learning are popular buzzwords that everyone seems to use nowadays. As shown in Figure 1, AI is a broader term than the other two. Machine learning includes algorithms for various kinds of task, such as regression, clustering, etc., and algorithms should be trained on data. The more data you provide to your algorithm, the better it gets. Deep learning is a very young field of artificial intelligence based on artificial neural networks. Deep learning algorithms also require data in order to learn to solve tasks.

Since AI-based technologies are now being integrated into daily life, applying AI-based technologies will be indispensable for every organization [3,5,24]. Even if deep learning has advanced in solving problems in AI areas for many years, organizations need to consider computational costs to be occurred in training the algorithms using the vast amount of data [25].

AI-based technologies are being integrated into our daily lives [5]. For example, the AI speaker “Aria” released by SK Telecom of South Korea is a smart voice-activated device [26]. Aria can make emergency calls when the bearer cannot use other devices because of accidents, physical handicaps, or unique situations. When an elderly person falls and says “Aria, please help,” Aria calls the care center, designated family member for emergencies, or ADT Caps (a security platform that provides machine security, personal guards, and security services in South Korea). If the center determines that it is, in fact, an emergency situation, it will report the incident to 119 (the emergency call number in South Korea). This support has already resulted in saving a number of elderly people who live alone. Aria can also be used as a recipe aid. If the user states, “Aria, please help me with a salmon recipe,” Aria will guide the user step-by-step through the recipe. Aria can even provide assistance with managing personal finances. Upon a user’s command, Aria can suggest the best credit card to use based on the interest rate and annual fees, or remind the user of the credit card payment due date in a given month.

Since AI-based technologies are now being integrated into daily life, applying AI-based technologies will be indispensable for every organization [3,5,24]. As smart mobile apps and devices become widely available in the digital age, consumers are demanding differentiated, personalized, and responsive services, with secure flexibility. In this context, it is important to analyze real-world cases of current AI use by healthcare organizations for patient care and management of operations, as well as to ascertain the requirements (e.g., regulation and responsibility) and support needs (e.g., ethics, training programs, or consulting services) for advanced AI applications.

### AI Applications in the Healthcare Industry

AI-based technologies are being integrated into our daily lives [5]. For example, the AI speaker “Aria” released by SK Telecom of South Korea is a smart voice-activated device [26]. Aria can make emergency calls when the bearer cannot use other devices because of accidents, physical handicaps, or unique situations. When an elderly person fell and says “Aria, please help,” Aria calls the care center, designated family members for emergencies, or ADT Caps (a security platform that provides machine security, personal guards, and security services in South Korea). If the center determines that it is in fact an emergency situation, it will report the incident to 119 (the emergency call number in South Korea). This support has already resulted in saving many elderly people who live alone. Aria can also be used as a recipe aid. If the user states, “Aria, please help me with a salmon recipe,” Aria will guide the user step-by-step through the recipe. Aria can even assist with managing personal finances. Upon a user’s command, Aria can suggest the best credit card to use based on the interest rate and annual fees, or remind the user of the credit card payment due date in a given each month.

Today, many startups (e.g., Freenome, an AI genomics biotech company in San Francisco; Recursion Pharmaceuticals in Salt Lake City; Benevolent AI in the UK; and OrCam in Israel, etc.) are providing healthcare solutions and services using AI-based technologies. IBM’s “Watson for Oncology” is the most widely utilized AI application in the healthcare industry, assisting assistance to doctors by suggesting appropriate treatment solutions. Mesko [11] proposed merging various systems with AI functions to develop healthcare apps that could be used to provide drug warnings, patient education material, and measurements of patients’ current health conditions. Moreover, Mesko [11] emphasized that AI-enabled devices, such as a personal assistant could significantly influence the monitoring and support of patients at times when medical staff are unavailable. AI-supported smart robots can also perform operations and augment physicians’ work with certain diagnoses, treatment methods, cost and time reduction, and improved response time to patients’ needs [3].

There are approximately 6000–8000 known rare diseases, with some 400 million people suffering from these diseases worldwide [27]. It is estimated that an average of five years is required to diagnose a rare disease [28]. Therefore, patients with rare diseases spend a great deal of time, efforts, and financial resources attempting to obtain an accurate diagnosis [27,28]. 3Billion, a bio-startup that provides DNA diagnosis services for rare diseases, reported that approximately 1200 patients have been diagnosed with rare diseases using AI. In suspected cases of the disease, 3Billion can test up to 7000 diseases at one time. One recently diagnosed patient received a different diagnosis from a doctor than what the AI-based technology suggested. CEO of 3Billion stated that “as the medical staff is not specialized in all diseases, they have to focus on just a few of the diseases they know. In the process, the patient can waste time engaging in ‘diagnosis wanderings’ from one hospital to another. This is a common problem for people with rare diseases all over the world. The number of patients that a doctor can take care of is limited. Working in the medical field AI can save hundreds of millions of lives around the world” [29].

Doctors at the Moorfields Eye Hospital in London have developed an AI diagnosis system, which can recommend treatments for more than 50 eye diseases with 94% accuracy [30]. In China, AI-based technologies are being used to diagnose colon polyps. In one clinical study, AI-based technologies and a gastroenterology specialist worked together to diagnose a patient, while in another clinical trial only a specialist was diagnosed; when diagnosed with the support of AI, the detection rate of polyps increased by 20% [31].

Researchers at the University of Southern California (USC) conducted an experiment using Ellie, a virtual human avatar. The results demonstrated that patients were more likely to tell their secrets to Ellie than to their close friends [32]. WeBot, a US startup that introduced an AI psychological counseling program, also reported similar research results. In May 2019, Eunpyeong St. Mary’s Hospital of the Catholic University of Korea introduced Robot Paul to accompany medical staff on rounds to inpatient rooms and introduced Robot Maria to guide customers to certain areas of the hospital. These two AI robots are equipped with self-driving, chatbot, and blockchain technology; Robot Paul also has voice electronic medical records (EMR) technology embedded [33].

IBM’s “Watson for Oncology” is the world’s first medical AI. It has a cloud-based AI platform for entering cancer patient data that helps medical staff devise treatment methods based on numerous past clinical cases, 290 medical journals, 200 textbooks, and 12 million pages of medical research [34]. However, in recent years, Watson has been criticized for its lack of accuracy (coincidence rate) and effectiveness in different medical fields [26,35]. It has been reported that large hospitals and medical staff in the US do not feel the need to adopt IBM Watson. Due to the nature of healthcare services, medical staff-patient workflows are important. Given that Watson is unable to interact organically between medical staff and patients, the US medical community is currently evaluating whether or not it is cost-effective [26,35].

The higher the consensus between Watson and medical staff on how to treat a patient, the more reliable Watson’s decisions are assumed to be. However, diagnosis results and the concordance rates differ depending on the patient’s unique characteristics and type of cancer involved. Regarding this issue, IBM stated that Watson may yield a low match rate due to variations in patient ethnicity, country-specific disease patterns, medical systems, and culture, and thus suggested that there is a need to improve the function and accuracy of AI by accumulating more data [22,35]. This means that AI-enabled technologies cannot replace all of a doctor’s functions and knowledge about individual patients’ care. However, despite these limitations, the expanded use of AI is undoubtedly creating a major change in the healthcare service market and the spread rate should increase as advances in AI accelerate in the future.

Miyashita and Brady [21] argued that AI may not generate the same benefits in the medical field as it does in general business fields. Although the application of AI may improve patient disease treatment outcomes, AI is not intended to fundamentally improve healthcare services or significantly reduce costs in the near future [21]. However, leading healthcare organizations, that endeavor to innovate ways to decentralize care units and optimize administration, continuously deploy AI-enabled technologies to do things that have never been done before. Miyashita and Brady [21] (p. 2) noted that “there is a wide array of non-acute health decisions that consumers make daily. These decisions do not warrant the attention of a skilled clinician but ultimately play an important role in determining patients’ health and ultimately the cost of healthcare.” Therefore, an expansive view of AI-enabled technology is necessary to consider its possible impacts on healthcare. 

## 3. Real-World AI Application Cases in Healthcare

According to the World Health Organization [36], 60% of factors related to individual health and quality of life correlate to lifestyle factors, such as exercise, diet, sleep, stress reduction, substance and medication abuse, and/or recreation [37]. AI-aided technologies and their applications can now provide lifestyle interventions and reminders during the day based on an individual’s vital signs through digital devices. Within healthcare organizations, AI-based technologies are set to significantly transform how healthcare systems operate, optimize, and interact with patients, and provide care services to increase the overall efficiency of patient outcomes.

### 3.1. Diagnostic Assistance

AI is expected to facilitate the diagnosis of patients with specific diseases. Taylor [38] (p. 1) reported that “diagnostic errors account for 60% of all medical errors and an estimated 40,000 to 80,000 deaths each year in U.S. hospitals.” Therefore, the use of AI-based technologies in various healthcare fields can help reduce errors made by human judgment [39]. 

The Mayo Clinic, a premier healthcare organization in the US noted for its innovation for patient care and health technology, employed AI for cervical cancer screening to identify pre-cancerous changes in a woman’s cervix. The AI-based solution uses an algorithm employing over 60,000 cervical images from the National Cancer Institute to identify precancerous signs. Researchers reported that the algorithm functions at a much higher accuracy rate (91%) than a trained human expert (69%) [40,41].

The Moorfields Eye Hospital in London, a specialist hospital of the National Health Service Foundation (NHSF), announced an AI solution to identify eye disease signs as effectively as world-leading doctors and experts. The AI-based solution assimilated collected data from over 15,000 British patients, allowing the algorithm to spot eye diseases from optical coherence tomography. The hospital announced that the AI-based system provided “the correct referral decision for over 50 eye diseases with 94% accuracy, matching world-leading eye experts.” Dr. Pearse Keane of Moorfields Eye Hospital noted that “the number of eye scans we’re performing is growing at a pace much faster than human experts are able to interpret them” [42]. This means that the assistance of AI-based technology can dramatically reduce diagnostic time.

At the Gachon University Gil Medical Center in South Korea, the ratio of consensus between medical staff and Watson was 55.9% in the evaluation of medical treatment results in one year (2017). However, the consensus rate for patients with stage IV stomach cancer was only 40%. Moreover, in April 2018, Konyang University Hospital in South Korea announced that the consensus rate between doctors’ decision-making and Watson’s treatment recommendation was 48%, based on 100 patients with breast cancer [26].

Liang et al. [12] examined the “evaluation and accurate diagnoses of pediatric diseases using AI” at the Guangzhou Women and Children’s Medical Center, which is a major academic medical referral center in Guangdong Province, China. The test applied AI-based technologies with deep learning techniques using 101 million data points from electronic records of 1.3 million outpatient visits to the medical center. To compare performance between the AI system and physicians, the study divided the physicians into five groups based on practice experience as follows: Group 1—senior resident physicians with more than three years of experience; Group 2—junior physicians with eight years of experience; Group 3—midlevel physicians with 15 years of experience; Group 4—attending physicians with 20 years of experience; and Group 5—senior attending physicians with more than 25 years’ experience. The AI-based model achieved an average accuracy score of 88.5%. This score was higher than that of the two junior physician groups (G1, 54.1% and G2, 83.9%) but lower than the three senior physician groups (G3, 90.7%; G4, 91.5%; G5, 92.3%). Liang et al. [12] (p. 436) suggested that this “AI model may potentially assist junior physicians in diagnoses but may not necessarily outperform experienced physicians.” In addition, the study concluded that the AI system was able to diagnose conditions with 90 to 95% accuracy rates. 

The Manifal Hospital, one of the top cancer care centers in Bangalore, India, introduced Watson for Oncology in 2015 and found a significant difference in diagnoses by the medical staff (multidisciplinary team) and Watson’s judgment using datasets of 1000 cancer patients, including those with breast cancer, colorectal cancer, rectal cancer, and lung cancer collected by two doctors over three-years. In the case of rectal cancer, the consensus rate between Watson’s treatment recommendation and doctors’ decisions was 85%, but the consensus rate for lung cancer was just 17.8%, demonstrating a large discrepancy between the two depending on the type of cancer [43].

At the Annual Meeting of the American Society of Clinical Oncology (ASCO) in 2019 in Chicago, Jeff Lenert, Associate Chief Medical Officer of Watson Health at IBM, reported that AI would assist scientists to make more informed decisions based on scientific evidence, and improve patient satisfaction by providing a comprehensive view of treatment options.

### 3.2. Nursing and Managerial Assistance

As is widely known, healthcare staff is often inundated with much paper work in the care process. This workload has prompted the industry to transition to electronic systems that integrate and digitize medical records, which is aided by AI-based technology. In addition, the use of chatbots has been identified as a potentially effective tool for engaging in conversation with patients and family members in hospitals [44].

The Cleveland Clinic, a nonprofit multispecialty academic medical center in Cleveland, Ohio, began using Microsoft’s AI digital assistant Cortana in 2016 to “identify potential at risk patients under ICU care” through predictive and advanced analytics. Cortana is integrated into Cleveland Clinic’s e-Hospital system and monitors “100 beds in 6 ICUs” from 7 p.m. to 7 a.m. An AI-assisted system of the University of Pittsburgh Medical Center can also listen and learn from conversations between doctors and patients in hospital rooms [45].

In March 2016, the Johns Hopkins University Hospital, a non-profit academic medical center in Baltimore, Maryland, announced a collaboration with GE healthcare partners to use predictive analytics based on AI technologies to support a more efficient operational flow [45]. The Johns Hopkins Hospital Command Center receives “500 messages per minute and integrates data from 14 different Johns Hopkins IT systems across 22 high-resolution, touch-screen enabled computer monitors.” James Scheulen, chief administrative officer for emergency services and capacity management at Johns Hopkins, reported that as a result of AI technology, “emergency room patients are assigned a bed 30% faster; transfer delays from operating rooms have been reduced by 70%; ambulances are dispatched 63 min sooner to pick up patients from other hospitals; and the ability to accept patients with complex medical conditions from other regional and national hospitals has improved by 60%” [45].

Other real-world examples that apply AI-based technologies in the healthcare system comprise Robotic-assisted Surgery and Virtual Nursing Assistants. Robotic-assisted surgery is preferred by surgeons due to its high precision, controllability, and flexibility. Robotic-assisted surgery can allow surgeons to perform surgeries that are very complicated or that were previously impossible. Advanced technological enhancements allow physicians to view additional patient-critical information in real-time even during surgery that combines real-time data with medical records, thus benefiting from AI technologies that leverage previously successful data regarding the same type of surgeries [39].

The Cedars-Sinai Hospital, a non-profit tertiary 958-bed hospital located in Los Angeles, California, employs Alexa robots developed by Amazon as virtual nursing assistants in inpatient rooms. Alexa completes nurses’ repetitive tasks to assist patients with their daily routines, reminds patients to take medications or to attend appointments, and helps answer medical questions [39]. 

At the Eunpyeong St. Mary’s Hospital of the Catholic University of Korea, the AI robot Paul assists medical staff on their rounds of patient visits and provides a list of inpatients to be treated by medical staff when a staff member scans a doctor’s ID card. The robot also accompanies medical staff to the ward, recognizes the voices of medical staff, and converts speech into text to transcribe electronic medical records in real-time. It also provides patient information, such as medical records, medical images, and test results, in real-time through the hospital system to assist the medical staff on their rounds. This robot can reduce the recording work of medical staff, accurately and quickly check patients’ medical examinations, and inspect information in real-time, as well as providing answers based on a real-time information-linked function through machine learning to enhance the efficiency of care services [33]. In the lobby of the hospital, the guide robot Maria provides customers directions to areas throughout the hospital. When a patient touches the robot using their own medical ID card, Maria guides them to the appointment schedule and the location of the doctor’s office. Maria can also guide patients to a particular medical department in the hospital [33].

The examples of AI-based diagnostics and administrative workflow assistance presented above highlight the expanding scope of AI to various areas in the healthcare system. To continuously expand and improve the quality of AI-supported systems, we examine some of the issues in the healthcare industry that need to be addressed.

## 4. Opportunities and Challenges Involved in AI Applications in Healthcare

To make AI-applied systems more accurate in making a diagnosis, the market should develop systems for each specialized area designed with machine learning algorithms with a sufficient number of cases that include ethnic and cultural information of patients [46]. Such AI systems can become smarter as more learning cases are added by healthcare researchers and practitioners. Just like any application of a new technology, the impact of medical AI systems has both utopian and dystopian aspects. The utopian perspective includes many new opportunities to treat diseases more effectively, provide better quality care and patient experience, encourage patients’ participation in the treatment process, reduce medical errors and healthcare costs, and improve the managerial efficiency of care providers [3,7]. The dystopian perspective, however, presents many new challenges that are daunting. The increased use of patient data for analytics can increase the cybersecurity risks for privacy and security [47], accountability of medical errors [22], and the possible impact on job loss [48]. We believe that some of the major positive and negative issues involved with the application of AI-based technologies should be examined to assure a smart use of AI and its wide diffusion in the healthcare industry. 

### 4.1. Opportunities with AI Applications

There is a wide spectrum of new opportunities gained through the expanded application of AI-based technologies in the healthcare industry. Some of the important ones are discussed here.

#### 4.1.1. Improved Disease Treatments

The introduction of IBM Watson was a momentous beginning of the age of data-based medical research which raised the social interest about the benefits of applications of advanced digital technologies to improve the public health and patient care quality [49]. As discussed in the real-world cases of AI applications in healthcare, advanced technologies are playing an increasingly important role in augmenting medical staff in almost every area of patient treatment. For example, Dawes et al. [50] reported how patients with high blood pressure and lung disease can be treated with more accurate data based on an AI-supported magnetic resonance imaging (MRI)-based algorithm of cardiac motion. In the same vein, 3Billion developed an algorithm to diagnose rare DNA based diseases in 2019. Guo and Li [51] reported AI-based technologies can greatly improve patient care services in rural farm communities of developing economies. 

AI has been proven to be especially effective with a large volume of radiology data to improve the quality of care services with medical imaging [52]. If AI-based software can improve the accuracy of patient diagnoses, then it will greatly help not only patients, but also the work of medical staff. For example, the frequency analysis of mitosis in cancer cells through images or microscope is a straightforward process, but takes a great deal of time. AI software can perform this task with greater accuracy and speed, thus, helping medical staff with their professional work while eliminating some of the drudgeries of tasks. AI-supported medical software can get smarter with learning from the increased volume of accumulated data and new medical research. This fact is proven as the increased accuracy of AI-supported medical software is approaching or exceeding the accuracy of medical experts in diagnosing diseases. The continuous research in the use of AI systems will greatly augment the work of medical staff as they can alert some areas that humans often miss or help minimize medical errors during the patient treatment. 

#### 4.1.2. Improved Patient Engagement and Participation

Noom, one of the most popular smartphone-based health coaching apps, is a diet app that fully functions as a mobile diabetes prevention program [53,54]. The company states, “we work with customers across the globe to help them create healthier habits, reduce their risk of chronic health problems, reverse disease, and foster healthier relationships with themselves in the process” [54]. The key to achieving the goals a person sets in using this coaching app, he/she must be fully committed to the program.

Patient participation in the medical treatment process is imperative for accurate disease diagnosis and patient safety. In addition, patients themselves perceive their personal participation in sessions with medical staff as a valuable and positive experience for their own sake [26,55,56]. When patients are encouraged to participate in their medical treatment, they tend to be fully engaged in carrying out their part in the process, which has a positive influence on their satisfaction with the care quality [26]. Boulding et al. [57] reported that patients’ positive experience of their engagement in the treatment process has positive impacts on the treatment result and patients’ safety. Therefore, to enrich the patient experience as a means to improve care quality, patient engagement and participation should be a strategic goal of healthcare providers [3,4].

While, patients may not be familiar with AI or AI-supported medical systems, they are more likely to participate in the system supported treatment process if they learned from the popular media or the attending physician about the possibility of faster and more accurate diagnosis, reduced medical errors, and decrease of the medical cost. With the rapid advances of AI and AI-imbedded medical systems, healthcare systems should develop strategies to inform and educate customers (patients and family members) about the merits and risks of the new systems. Well-informed customers will more willingly participate in the use of AI medical systems, and thus, increase the flexibility of their treatment options.

#### 4.1.3. Improved Medical Error Reduction and Service Quality

Wang et al. [31] reported that, in China, doctors who performed colonoscopy examinations with the support of AI discovered 20% more polyps than those without. The AI-supported system can find the notoriously small (5 mm or less in size) or early developmental polyps that many gastroenterologists miss during the colonoscopy exams. Therefore, AI systems augment doctors in eliminating problematic small polyps that can cause future problems, improving care service, and reducing medical error possibilities.

A research team at the University of Tokyo Medical School reported the development of an AI system based on new algorithms and order parameters. When this system was integrated with a deep-learning AI medical program, the highest accuracy rate found was 83.5% when applied to a sample patient group. However, after the system was interfaced with a deep-learning and decision tree AI system, the accuracy rate increased to 87.3% [58]. Recently developed smart AI systems can further reduce the error rate and they are expected to further improve the care service quality.

Radiologists are often cited as the most likely medical personnel who will be displaced by AI. This prediction is based on the fact that a radiologist can read 50–100 X-rays a day, while an AI-supported system can read 10–100 times more images [52]. In addition, the accuracy of the AI system is superior to that of radiologists. Thus, when the AI system augments radiologists, doctors can utilize the time saved by the AI system to conduct more friendly and meaningful discussions with patients that can help improve the quality of care service. In addition, with the more accurate data extracted by AI, medical staff can prevent possible medical errors in advance.

#### 4.1.4. Improved Operational Efficiency and Reduction of Medical Cost

AI-supported medical systems, as discussed above, can handle many diagnostic activities without human intervention. For example, an AI-imbedded pill-cam can replace laborious traditional upper endoscopy to check stomach cancer exam [51]. Escalante et al. [59] reported a new AI-based method to examine the bone marrow structure characteristics to test the acute leukemia, which can replace the high cost conventional methods. These AI systems all help make the diagnosis and treatment processes much more efficient and cost effective.

AI systems are not exclusively for medical purposes. Some AI systems are designed to support operational innovations to create additional or new value in the value chain of a healthcare organization. AI systems can perform routine operational activities much better and faster than human workers, such as managing maintenance systems, accounting, and information inquiry. AI-enabled chatbots and nursing robots can greatly improve the efficiency of operational processes.

#### 4.1.5. Increased Productivity and New Job Creation

Will robots and AI take over everything that humans are doing currently? The history and evolution of industrial development, from the 1st Industrial Revolution to the fourth Industrial Revolution, have shown that while many routine manual jobs were replaced by technologies, many new jobs have also been created to support productivity increase [60]. For example, although the hard-copy printing business has diminished greatly, many new jobs have been created in digital editing and typography [61]. Many map publishers have closed their doors, on the other hand, numerous new jobs were created to develop navigation and geographic information systems.

Javanmardian and Lingampally [62] articulated the role of AI in productivity improvement through higher efficiencies in healthcare: “AI solutions are already producing the kind of internal gains that suggest much more is possible in healthcare players’ back offices.” In the case we discussed earlier, Noom had 77 employees in 2017, but the number increased to 1100 by June 2019. While, the AI-enabled app Noom helps people live with healthy lifestyles, from managing their dieting routines to preventing long-term diseases, the AI system was able to detect the reasons why some customers quit the system use and not able to fulfill their original goals. Therefore, the company introduced Noom Coach to teach people that health management requires not only regular and disciplinary diet and exercises but also psychological motivation. Thus, Noom Coach began to provide customized one-to-one support services to maintain sustained relationships with the customers to help them attain their health goals [54]. In other words, Noom recognized through the AI system that technology alone cannot change people’s behavior, but psychological analysis of emotions and desires should be incorporated to make the system work. In the process, Noom has been able to create many new jobs.

Another good example of the increased productivity increase would be the AI-enabled eye disease diagnosis system for macular degeneration and diabetic macular edema through transfer learning developed in collaboration between San Diego State University and China’s Guangzhou University Medical School [63]. This AI system can complete the diagnosis process of identifying the disease and its stage of development in just 30 s. Furthermore, the accuracy rate of diagnosis, in comparison with the collective diagnosis of five expert ophthalmologists, was over 95%. These examples clearly indicate that AI-based systems can improve productivity by decreasing the error ratio, saving the diagnosis and treatment time, and exploring opportunities to expand care services that were not possible in the past. 

#### 4.1.6. Reduced Healthcare Cost

The ideal healthcare service would include the following: data and evidence-based disease prevention, diagnosis and treatment with the best available technologies, patient-centric customized care, and quality care with empathy from medical staff [3,4]. If AI can be applied broadly to support such ideal care service, then it can help secure both quality care service and significant savings in medical costs. According to a report by ABI Research, a consulting firm for marketing research, smart applications of AI in the healthcare industry can save as much as $52 billion by 2021 in the US [64]. ABI Research also stated that major hospitals in the US and Israel already use AI-based programs for disease prevention. The number of AI-supported devices for patient training to prevent chronic diseases (e.g., diabetes, high blood pressure) in these two countries is expected to increase from 53,000 in 2017 to over 3.1 million by 2021, an annual increase of 176%. Thus, AI applications in healthcare can be a major force for reducing medical costs, not only for individual patients but also for society at large. At the national level, such savings can be diverted to prevention of diseases for better quality of life of all citizens.

### 4.2. Challenges Involved with AI Applications

While AI applications offer new opportunities to improve people’s daily life, it also brings challenges that must be managed effectively. The challenges are especially daunting in the healthcare sector because human lives are at stake. Some of the challenges that need to be managed with wisdom are as follows. 

#### 4.2.1. Accountability of the System Use

When a Tesla Model S autonomous car had a malfunction and killed a person on 7 May 2016, a major question had to be answered “Who should be accountable for the accident?” If medical staff used AI-based technologies for patient treatment and an accident or error occurred, then who should take responsibility for this outcome? AI-based programs operate based on a set of machine learning algorithms designed by people. Hospital administrators, with the help of technological experts or consultants and medical staff, decided to purchase the system for its wide-ranging application potential. Medical staff at a hospital applied the system to provide needed care services for a patient. Then, should the responsibility of the problem be on the system design firm, hospital, or medical staff? This is a very difficult question to answer as it involves a number of technical, managerial, and ethical issues.

Although AI-related technologies are advancing rapidly and their applications are widespread, there has been relatively little research done on the ethical aspects of AI. Dr. Stephen Hawking observed that with the rapid growth of AI and smart robots, the world is fast approaching a point where human power will be out of control. He also suggested that we must establish a new global governance agency to regulate the use of AI [65]. Lupton [66] emphasized that moral and ethical behavior patterns be developed for AI in a way that is positive rather than negative for society. As AI-based technologies/systems in various fields are likely to expand greatly in the future, they should be designed so that their performance with humans be aligned according to social norms and values. Especially in the transformative healthcare industry, accountability of any negative consequence of AI applications should be based on social agreements.

#### 4.2.2. AI Divide

One characteristic that distinguishes the healthcare industry from other service industries is that patients often trust medical staff unconditionally [67]. This can be explained by the placebo effect; it has been reported that when a patient blindly trusts a doctor’s treatment and believes that his/her illness will be cured, a medical effect occurs [67]. In other words, physician-patient trust is vital because it helps improve the effectiveness of care treatment. If an AI-based technology/system assumes the role of a doctor, then the patient will be participating in a care delivery process that builds a relationship with an artificial system rather than with a human doctor. The success of this new relationship between an AI-enabled technology/system and the patient also depends on trust [66]. However, people who have not had any experience with digital technologies, let alone AI, would have much difficulty trusting an AI system. This AI divide can possibly be overcome if the doctor can assure the patient how the system will help him/her to receive better care.

#### 4.2.3. Cybersecurity for Privacy and Security

Since AI-based technologies/systems are based on vast datasets, privacy issues arise in terms of data collection and sharing [9,19]. Disease-related data are very difficult to share and regulate across diverse databases because patient records contain personal information [68]. This means that companies that develop software must adhere to confidentiality rules, which can lead to obstacles in AI development. As AI technology reaches possible conclusions based on the machine learning of the accumulated data provided, the decision-making process does not consider the specific circumstances of individual patients, which raises ethical, moral, and legal issues. Thus, it is necessary to discuss the rules and norms that AI technology should observe, such as ethics, laws, and personal values which regulate individuals’ behavior in society. 

#### 4.2.4. Loss of Managerial Control

In the digital age, many industries are no longer isolated silos. The healthcare industry is no exception. In the past, the healthcare sector used to be considered a place where patients get their diseases treated by doctors and nurses. However, today good health is the consequence of healthy living habits like a good diet, regular exercise, and daily well-being routines, in addition to quality medical services. Thus, preventive medicine has become important, and in the process, the boundaries of a healthy lifestyle, medicine, and technological support have become very blurred [64]. AI can surpass time and spatial barriers. As such, the traditional concept of a closed healthcare system is no longer relevant. For example, there are many smartphone-based apps (e.g., Robot Maria, Alexa, AI speaker Aria, etc.) that can help the integration of different aspects of people’s well-being. AI-based systems monitor, diagnose, treat, and manage home-based patients remotely.

With the widespread application of AI technologies in the healthcare industry, care providers are increasingly dependent on the expertise of consultants, specialists, and experts in ICT, convergence, and human resource management. Therefore, care services have become a team sport of many external and internal experts. Consequently, the traditional bureaucratic governance system would not work in modern hospitals. In the process, hospital administrators may sense the loss of managerial control. However, this is a new form of governance, a dynamic living system that integrates and connects every system, device, and person to provide the best possible care to the patient [5].

#### 4.2.5. Job Loss, Training/Education Needs, and the Pain of Transformation

Recently, Amazon announced that it would re-educate 100,000 employees through training programs on new technologies by 2025 to prepare for more highly skilled jobs in the AI era. Jeff Wilke, Amazon’s worldwide consumer CEO, stated that “as technology changes work, they have the opportunity to advance in their career and take advantage of those changes” [69]. Another example is the Health Innovation Big Data Center at Asan Medical Center in Seoul, Korea which just initiated education programs for future AI experts who can develop and commercialize AI algorithms [70].

As discussed earlier, many believe that radiology is a medical specialty that may disappear in the future because AI will be able to analyze diagnostic medical images more accurately than humans. However, it is argued that the introduction of AI will allow radiologists to provide more specialized diagnostic services to patients [52,71]. It is certain, however, that AI-related technologies will make many repetitive jobs obsolete. While some jobs will be lost, many new jobs will be created to support the implementation of AI-based systems and devices.

In the long run, AI is expected to allow for deeper relationships between healthcare providers and patients by, for example, supplementing AI’s shortcomings. For this reason, medical school curricula should effectively utilize AI-related education and technology. In addition, more accurate AI applications should be developed because medical AI is limited by historical datasets of patients. Of course, this development requires the positive participation of medical staff at the beginning of the AI development stage.

To address this issue, it is necessary to foster a highly-trained professional team that can respond rapidly in the consumer-oriented healthcare market by providing diverse learning opportunities related to advanced technologies, such as using AI to collaborate with medical staff. We believe that new curriculums (e.g., technology innovation and application, human-machine convergence, data analytics courses, human-machine interchange, cyber ethics, and responsibility, etc.) should be added to the medical school curriculum to create new jobs. 

## 5. Conclusions

Innovation is imperative in the dynamic digital age. The rapidly advancing technologies are the primary tools for implementing and converging value-creating ideas [5]. Therefore, the application of AI and related technologies is not a choice, but a trend that organizations must accept and leverage for competitive advantage. The healthcare industry is especially susceptible to the new application potential of AI because of the transformative nature of healthcare. AI applications are not only changing the nature of care delivery in terms of the diagnosis and treatment processes but also the lifestyle of patients themselves as their well-being requires the entire package of healthy living routines. 

In this study, we have examined how AI technology has been diffused in healthcare and the type of new opportunities and challenges it has brought. Managing these opportunities and challenges effectively requires the collective wisdom and resolve of all the stakeholders of the healthcare industry.

### 5.1. Implications of AI Applications in Healthcare

Advances in digital technologies have greatly broadened their application areas. Furthermore, another important aspect of such advances is the increased ease of use and usefulness of digital technologies [7]. Therefore, while deep and broad, the digital divide (also the AI divide) is narrowing and consumer behavior has been changing dramatically across industries. Healthcare service is no longer an isolated silo with the monopolistic power of the medical staff. Patients’ health is the result of many contributing factors: healthy diet, regular exercise routines, management of emotional and psychological stress, preventive medicine, and of course cure of diseases. Today, many patients do their own research about their health or ailment conditions through online services. In addition, AI and other digital devices are widely used to augment the work of medical staff. Thus, a holistic view of the dynamic healthcare environment is needed to examine the trends of new technology applications, as well as the opportunities and challenges that these changes bring to bear. This study focused on innovations in the healthcare sector based on AI. The results of this study should shed new insights to researchers and practitioners of healthcare about the magnitude of application potential of new technologies, especially AI in their care delivery and operations. 

For the application of AI-based systems, it is important to collect and analyze data of various types (e.g., including ethnic and cultural characteristics of patients) because machine learning algorithms of the systems require a sufficient volume of data for an accurate diagnosis. While there are positive and negative issues involved with the application of AI and its various aspects, it is a reality that AI has made a significant inroad into the healthcare sector and this trend is expected to accelerate in the future [9,62]. Thus, it is necessary to increase the research, accessibility, and actual use of AI in the healthcare industry. We would like to suggest several approaches to best manage AI applications in healthcare as follows. 

First, it is necessary to establish a legal framework for information access and sharing for AI applications. A real-time data acquisition of information and sharing is required to enhance AI performance in healthcare. The quality of data is very important because “the better the data quality, the more confidence users will have in the outputs they produce, lowering risk in the outcomes and increasing efficiency” [72]. Johnson et al. [73] suggested that healthcare organizations should prioritize data quality improvement efforts for validity and assess whether the values that are reported should be trusted. The qualitative accumulation of accurate and realistic data is vital because patterns of disease vary depending on individual characteristics such as ethnic and cultural background, lifestyle, socio-economic conditions of the patient’s living area, etc. Therefore, to improve the accessibility of high-quality data at the governmental level, reasonable data access criteria and systems, with the consideration of risks and benefits of data sharing, should be established. In addition, legal standards are required to protect each patient’s personal information and to use such data solely in a collective form for public purposes.

Second, social consensus must be reached for the critical aspects of AI, including data sharing confidentiality, and liability. To acquire data, which is the essential resource of medical AI, the participation of general public should be encouraged. In addition, social consensus is required on the quality management of AI-based systems and liability for possible misdiagnosis or medical accidents during the care service [74]. Although there has been a great deal of concern about leakage of personal information, no social consensus has yet been developed on data ownership or sharing and technology produced medical errors. 

Third, applications of AI-based systems require the collaboration of specialists in several related areas for care service. This point has been stated clearly by a report: “AI is like a surgical knife in the end, and how closely we work with real-world experts in healthcare fields will be the success or failure of the medical AI solution, and from the development stage, nurses, doctors, social workers, pharmacists, and patients should work closely together to analyze and evaluate which technologies will be focused on developing” [23]. Therefore, in the development–application–analysis phases of AI application, a collaborative environment must be developed.

Fourth, instead of focusing solely on job destroying possibilities of AI systems, efforts should be made to best utilize surplus manpower in new areas of value creation. Although automation will result in machines conducting some tasks that humans have previously performed, automation will result in new types of work, similar to what occurred during the Industrial Revolution. Healthcare organizations should take the opportunity to transition displaced employees into new and more valuable work positions. For example, the number of radiologists may decline if AI-based systems use becomes prevalent in diagnosis and medical image analysis. Then, radiologists can transition into new positions as medical technologists, those who can converge medical science and ICT, much more valuable people in hospitals [52,71]. Last, the medical community, the ICT industry, and the government should cooperate to research on medical informatics to develop a platform for analyzing and sharing healthcare big data.

To enhance the utilization of AI and to invoke patients’ confidence in the healthcare system, several additional considerations should be explored, which are beyond the scope of this study, as follows. First, AI education should be required of most college majors, including the medical school (e.g., the MIT (Massachusetts Institute of Technology) Computer Science and Artificial Intelligence Lab is providing the required AI course for all majors at MIT, https://www.csail.mit.edu/) as digital devices are increasingly used in every industry. This new trend should be reflected in the training of future physicians in medical schools.

Second, advanced technologies can save time and cost, encourage patient participation in the use of technology-supported systems provided by healthcare organizations. In particular, AI can provide easy access to medical care, diagnosis, and monitoring outside of hospitals. Therefore, it is necessary to provide technologies/systems that are easily accessible and easy to use by the general public. Thus, education and training opportunities for patients and medical staff for AI and related technologies should be available to increase system utilization.

Lastly, information protection systems should be further strengthened to avoid the leakage of patient data through cyberattacks or due to operational errors in healthcare institutions. The assurance of cybersecurity will help promote the agreement of patients for using their medical data for public purposes. To facilitate this, an effective data security system and a moral responsibility agreement should be developed.

### 5.2. Limitations and Future Research Needs

The 5G network is expected to further spread the utilization of AI in the healthcare service market in the near future. For example, medical imaging devices, such as MRI, generate very large capacity files, and 5G networks can improve the quality and accessibility of care services by allowing large-capacity medical image data to be transmitted quickly and accurately. Since the 5G network can be used for telemedicine on networks without a LAN connection, patients can receive medical treatment or consult with specialists with speed. Ericsson [75], a world leader in communications technology and services, has predicted that the 5G market for healthcare services will provide a revenue opportunity of approximately $76 billion by 2026.

Responding quickly is the most effective way to minimize damage in situations where it is difficult to predict the future. Beyond time and space limitations, AI-based technology, which can surpass the limits of expert knowledge, is breaking the boundaries of healthcare, well-being, and life itself [2,74]. In this regard, although AI-based medical systems focus on patient-centered management of diseases, it is expected that, in the long term, the scope will expand from pre-disease to treatment and post-care to daily life. Therefore, AI-enabled technology is penetrating not only all aspects of healthcare but also our daily lives, and it should be applied using a comprehensive perspective that examines entire spectrum of people’s daily life.

The suggestions presented in this study are based on the current use of AI-based technologies, which may limit our understanding of the full potential of future technologies. Through a review of the literature and real-world applications of AI systems in healthcare organizations, this study has provided some directions for effective use and management of AI. We hope our study will stimulate more rigorous theoretical and empirical research for the most effective application of AI systems to ensure the best possible care to patients and provide preventive public health.

## Data Availability

Not applicable.
